# Reliability of CT attenuation value for adrenal masses

**DOI:** 10.1530/EC-25-0671

**Published:** 2026-02-20

**Authors:** J Yanagida, Y Yoshida, M Otsuki, S Sakai, Y Nagashima, K Horiuchi, T Okamoto

**Affiliations:** ^1^ Department of Endocrine Surgery, Tokyo Women’s Medical University, Tokyo, Japan; ^2^ Department of Endocrinology, Tokyo Women’s Medical University, Tokyo, Japan; ^3^ Department of Diagnostic Imaging and Nuclear Medicine, Tokyo Women’s Medical University, Tokyo, Japan; ^4^ Department of Surgical Pathology, Tokyo Women’s Medical University, Tokyo, Japan

**Keywords:** adrenal mass, CT attenuation value, reliability, hormonal activity

## Abstract

**Objective:**

CT attenuation value is useful in the differential diagnosis of adrenal masses, and values <10 Hounsfield units (HU) can exclude malignancy and pheochromocytoma. However, few reports have examined the reliability of CT attenuation measurements. We examined the reliability of CT attenuation measurements made by surgeons not specialized in image reading.

**Design:**

A retrospective analysis of 313 patients (325 lesions) who underwent surgery at a single institution was performed.

**Methods:**

One general surgeon and one endocrine surgeon independently measured CT attenuation values. The intraclass correlation coefficient and Cohen’s kappa coefficient were used to analyze interobserver reliability. All cases were subjected for endocrine function tests and histopathologically diagnosed. Additional analyses included segmented regression for size-related measurement variability and multivariable analyses comparing aldosterone-producing adenomas (APAs) and cortisol-producing adenomas (CPAs).

**Results:**

The intraclass correlation coefficient between observers was 0.938 (95% CI: 0.923–0.949), and Cohen’s kappa coefficient was 0.851 (95% CI: 0.781–0.922). Both observers measured all malignant adrenal tumors (such as adrenocortical carcinoma, metastatic adrenal carcinoma, and malignant lymphoma) and pheochromocytomas as ≥ 10 HU. CT attenuation values were significantly higher in CPAs than in APAs, independent of mass size (*P* < 0.001 for both observers).

**Conclusions:**

CT attenuation value was shown to be reliable enough for simple measurements that could be performed by non-radiologists and was useful for excluding malignancy and pheochromocytoma.

**Significance statement:**

This study reinforced evidence for the reliability of CT attenuation value. Even with a simple method that can be performed by non-radiologists, CT attenuation values were highly reliable and useful for excluding malignancy and pheochromocytoma. In addition, all lesions in this study were evaluated both endocrinologically and pathologically, giving high validity to the reference standard. These findings complement and further substantiate the latest clinical practice guidelines of the European Society of Endocrinology.

## Introduction

Computed tomography (CT) is a quick and easy way to screen for diseases and is widely used in clinical practice. However, various abnormal findings are often obtained incidentally. The frequency of finding an incidental adrenal mass on abdominal CT is about 4% (1). Adrenal mass found incidentally on imaging for purposes unrelated to the adrenal gland is called adrenal incidentaloma (AI). Ichijo *et al.* tabulated 3,972 cases of AI in Japan from 1999 to 2004 (2). In the same report, 50.8% of AIs were nonfunctioning adenomas, while lesions requiring treatment, such as aldosterone-producing adenomas (APAs), cortisol-producing adenomas (CPAs), pheochromocytomas (PCCs), and adrenocortical carcinomas (ACCs), accounted for 5.1, 10.5, 8.5, and 1.4% of AIs, respectively. The ability to reach a diagnosis from CT findings is highly desirable in order to avoid unnecessary examinations and follow-up for nonfunctioning adenomas.

The CT attenuation value reflects tissue density. Benign adenomas and myelolipomas are considered to be lipid-rich, resulting in low values, while ACCs and PCCs are lipid-poor, resulting in high values. Based on previous studies reporting that a cutoff value of 10 Hounsfield units (HU) or higher offers 100% sensitivity for diagnosing malignant adrenal masses, the guidelines of the European Society of Endocrinology and the European Network for the Study of Adrenal Tumors recommend no further testing for masses with a CT attenuation value less than 10 HU (3, 4, 5, 6). A systematic review on PCC also states that it can be excluded when the CT attenuation value is 10 HU or less (7). However, many of the studies included in those systematic reviews did not analyze the interobserver reliability of measurements (8, 9, 10, 11, 12).

A few studies have attempted to evaluate the reliability of CT attenuation value measurements. A retrospective study of 197 cases from France showed high reliability of CT attenuation value measured by two expert radiologists (ICC, 0.96; 95% CI: 0.94–0.97) (13). In a Dutch multicenter study, 222 PCC lesions were measured by two radiologists, with only one lesion <10 HU, and the interobserver reliability was high (ICC, 0.81; 95% CI: 0.75–0.86) (14). However, these studies primarily involved radiologists. Given that CT attenuation value measurement is a simple and rapid technique, extending its use to non-radiologists could facilitate faster clinical decision-making, and having both radiologists and surgeons perform the measurements may provide a double-checking function that enhances diagnostic quality. Accordingly, we sought to assess the reliability of CT attenuation measurements performed by surgeons.

The aim of the present study was to evaluate three aspects of CT attenuation value measurements independently determined by two surgeons: i) reliability, defined as the reproducibility of CT attenuation value readings; ii) interobserver agreement in the dichotomous classification (i.e., benign vs malignant) using a cutoff value of 10 HU; and iii) the validity of this classification for excluding malignancy and PCC.

## Materials and methods

### Study population

We retrospectively identified 338 patients who underwent adrenalectomy at our department from January 2008 to December 2022. Of these, 25 patients were excluded from the analysis because they did not undergo CT or CT did not show a mass. As a result, 313 patients (325 lesions) were included in this study.

### Measurement of HU using non-enhanced CT

**Figure 1:**
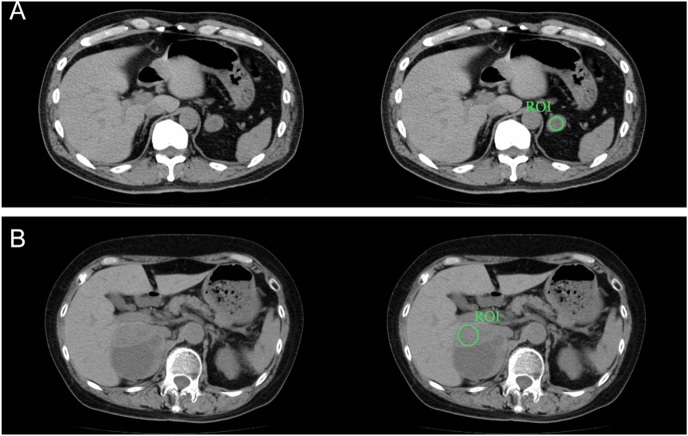
Simple rules for placing the region of interest (ROI). (A) In the case of a mass with a homogeneous interior, a circular ROI is placed to include the widest possible area of the mass. (B) In masses with a cystic component, the ROI is placed so that the largest circle can be drawn in the area without the cystic component. The same process is applied for calcification. Alt text: axial CT images showing methods for placing regions of interest (ROIs). (A) ROI placed to encompass the widest possible area within a homogeneous mass. (B) ROI placed to exclude cystic components and calcifications.

CTs included those performed not only in our hospital but also in other hospitals, so equipment and protocols varied. The obtained CT data were viewed using image viewing software (ShadeQuest/ViewR-DG; FUJIFILM, Japan) and analyzed by two independent observers (a surgeon with 9 years of experience and an endocrine surgeon with 16 years of experience). At the time of the study, the surgeon was undergoing training in endocrine surgery and performed approximately 2–3 adrenal surgeries per year, whereas the endocrine surgeon routinely performed approximately 20–30 adrenal surgeries annually and was also directly involved in the imaging assessment of AIs and the evaluation of endocrine function. Neither observer had formal radiology training. One observer measured CT attenuation value and mass diameter, and the other measured CT attenuation value only. Observers were blinded to preoperative diagnosis and postoperative histopathology. Measurements were taken on the slice with the largest cross-sectional area in the transverse section of the adrenal mass. A region of interest (ROI) was placed on the largest possible cross-sectional area of a mass in a large circle, but obvious cystic areas and calcifications were avoided (Fig. 1). In instances where multiple nodules were identified within a single adrenal gland, the largest mass was documented for measurement. Before initiating formal measurements, the observers conducted a consensus discussion using practice cases to align their measurements according to the predefined ROI placement rules.

### Data extraction

We retrospectively extracted data on age, sex, hormonal activity, and pathological diagnosis for each patient from medical records. The functional profile of adrenal masses was classified according to Japanese guidelines into nonfunctioning masses, aldosterone-producing masses, cortisol-producing masses (including Cushing’s syndrome and subclinical Cushing’s syndrome), and PCCs (15, 16, 17, 18). Masses with more than one type of hormone hypersecretion were considered composite types.

### Reference standard

For the reference standard, histopathological findings were used to determine mass malignancy and tissue origin (cortex vs medulla), whereas biochemical testing was used as the gold standard for assessing hormonal activity.

### Statistical analysis

The intraclass correlation coefficient (ICC) was used to analyze the interobserver reliability of CT attenuation value. A two-way random effects model with absolute agreement and single measurement was applied, corresponding to ICC (2,1). The ICC was computed using the following formula:

$$\text{ICC}\left(2,\;1\right)=\;\frac{MS_{R}-MS_{E}}{MS_{R}+\left(k-1\right)MS_{E}+\;\frac{k\left(MS_{C}-MS_{E}\right)}{n}}$$

where *MS_R_* is the mean square for subjects, *MS_C_* is the mean square for raters, *MS_E_* is the mean square error, *k* is the number of raters, and *n* is the number of subjects. ICC values were interpreted as follows: 0.0–0.5, poor; 0.5–0.75, moderate reliability; 0.75–0.9, good reliability; and 0.9–1.0, excellent reliability (19). Diagnostic concordance was evaluated using Cohen’s kappa coefficient (*κ*) and the percentage of exact agreement when 10 HU was used as the cutoff. *κ* was calculated using the following formula:

$$\kappa =\;\frac{Po-Pe}{1-Pe}$$

where *Po* is the observed proportion of agreement and *Pe* is the expected agreement by chance. *κ* value was interpreted as follows: 0.00–0.20, slight agreement; 0.21–0.40, fair agreement; 0.41–0.60, moderate agreement; 0.61–0.80, substantial agreement; and 0.81–1.00, almost perfect agreement (20).

To further examine factors affecting measurement variability, we performed segmented regression analysis using mass size as a continuous variable and the absolute difference in CT attenuation between the two observers as the dependent variable. For clinical applicability, masses were then categorized into seven size groups (≤15, 16–20, 21–25, 26–30, 31–35, 36–40, and >40 mm), and ICC values were calculated for each category. Four lesions (two malignant lymphomas and two hyperplasias) were excluded because indistinct margins prevented reliable size measurement.

In addition, the distribution of CT attenuation values for each disease is shown (Figs 3 and 4). To evaluate differences in CT attenuation between APAs and CPAs, we performed a multivariable linear regression analysis with CT attenuation as the dependent variable and mass size and hormonal activity (APAs vs CPAs) as independent variables. Significance was set at the level of *P* < 0.05. JAMOVI v2.6.45.0 software (jamovi project, Australia) was used to calculate ICCs and perform the segmented regression analysis and the multivariate linear regression analysis, and JMP Pro v17.0.0 (SAS Institute Inc., USA) was used to calculate *κ*.

## Results

### Patient characteristics

Table 1 shows the background of the 313 patients (325 lesions). The median age was 48 years, patients were predominantly female, and most patients had functional masses. Benign nodules include 175 adenomas, 9 hyperplasias, 10 neurogenic tumors, 1 neuroendocrine tumor, 1 hemangioma, 1 atypical adenoma, 1 hematoma, and 1 cyst. Three of the benign nodules (marked as ‘Other lesions’ in Table 1) showed catecholamine hypersecretion, which was considered preoperatively diagnostic for PCC, but the pathological findings led to a diagnosis of neurogenic tumor, neuroendocrine tumor, and hyperplasia in 1 case each.

**Table 1:** Background characteristics and CT attenuation values for each disease.

	Benign					Malignant				
	NFT	PA	CS, SCS	Composite type	Other lesions	PCC	ACC	Meta	ML	Total
*n*	17 (5)	75 (23)	94 (29)	10 (3)	3 (1)	115 (35)	6 (2)	2 (1)	3 (1)	325
Age, years	54 (37–69.5)	49 (41–56)	51 (42.75–63)	53 (47–66.5)	63 (59–80)	40 (33–56)	60 (53–71)	67.5 (63–72)	77 (60–78)	48 (38–61)
Men/women	7/10	33/42	19/75	1/9	1/2	49/66	0/6	2/0	1/2	113/212
Size, mm	40.9 (26.7–60.9)	16.5 (12.9–18.9)	28.7 (25.2–32.8)	19.2 (14.9–24.1)	27.6 (19–57.2)	38.2 (27.7–49.1)	52.9 (27.4–89.9)	53 (19.9–86.1)	46.4 (46.4–46.4)	28.15 (19.7–38.3)
CT attenuation, HU										
- Observer 1	32.1 (27.1–41.4)	8.4 (1.6–15)	20.3 (11.0–31.9)	35.5 (24.6–41.5)	25 (4.3–26.4)	42.3 (34.8–46.2)	37.6 (28.7–41.5)	32.3 (27–37.6)	37.3 (34.7–43.2)	29.3 (12.3–40)
- Observer 2	30.5 (26.3–41.5)	8.1 (1.1–15.5)	20.2 (10.9–30.7)	36.6 (24.3–40.3)	25.7 (5.8–27.3)	41.5 (35–46.3)	37.5 (29.2–38.9)	30.4 (23.8–37)	35.4 (33.5–35.8)	28.6 (12.5–39.2)

Data are expressed as median and interquartile range (IQR) or number and percentage.

NF, nonfunctioning mass; PA, primary aldosteronism; CS, Cushing’s syndrome; SCS, subclinical Cushing’s syndrome; PCC, pheochromocytoma; ACC, adrenocortical carcinoma; Meta, metastatic adrenal carcinoma; ML, malignant lymphoma; and HU, Hounsfield units.

Alt text: Table 1 summarizing the background of 313 patients with 325 adrenal lesions. Median age was 48 years, with a female predominance, and most lesions were functional masses.

### Reliability of CT readings

**Figure 2:**
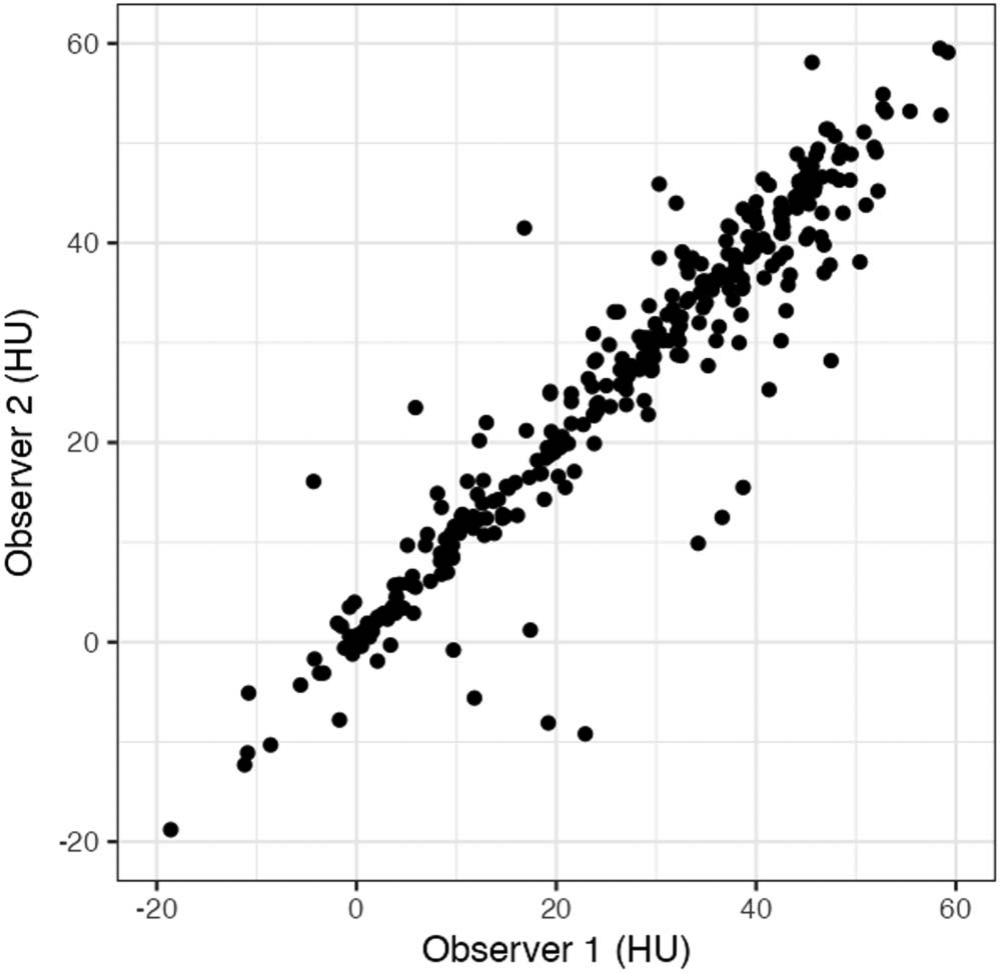
Interobserver reliability of CT attenuation value. The intraclass correlation coefficient (ICC) is 0.938 (95% CI: 0.923–0.949). Alt text: scatter plot of CT attenuation values measured by two observers. It shows a strong correlation between observers.

Median measured CT values for observers 1 and 2 were 29.3 HU (interquartile range (IQR), 12.3–40.0) and 28.6 HU (IQR, 12.4–39.2), respectively. Figure 2 shows the interobserver reliability. The ICC of the CT value was 0.938 (95% CI: 0.923–0.949). Segmented regression analysis using mass size as a continuous variable and the absolute interobserver difference as the dependent variable revealed a tendency for measurement error to increase when mass size was ≤16.6 mm. When ICCs were calculated across seven mass-size categories, the lowest reliability was observed in the ≤15 mm group, followed by another decrease in the >40 mm group (Table 2).

### Observer agreement of diagnostic judgment

Table 3 shows the observer agreement of diagnostic judgment. Diagnostic concordance with a diagnosis of ≥10 HU or <10 HU as measured by *κ* was 0.851 (95% CI: 0.781–0.922).

**Table 2:** Interobserver reliability across mass size categories.

Size category (mm)	*n*	ICC (2.1), 95% CI
≤15	31	0.808 (0.637–0.903)
16–20	54	0.949 (0.914–0.970)
21–25	40	0.991 (0.984–0.995)
26–30	59	0.894 (0.828–0.936)
31–35	31	0.953 (0.906–0.977)
36–40	32	0.884 (0.777–0.942)
>40	74	0.843 (0.762–0.898)

Intraclass correlation coefficient (ICC) was analyzed after stratifying masses into seven size categories (≤15, 16–20, 21–25, 26–30, 31–35, 36–40, and >40 mm). ICC was lowest in the smallest masses (≤15 mm), and a decrease in reliability was also observed in the largest group (>40 mm).

Alt text: Table 3 comparing interobserver reliability across seven mass size categories, showing the lowest reliability in ≤15 mm and >40 mm masses.

**Table 3:** Diagnostic concordance between observers.

		Observer 2		
		≥10 HU	<10 HU	Total
Observer 1	≥10 HU	249	6	255
	<10 HU	10	60	70
	Total	259	66	325

This table shows the diagnostic results for each observer when 10 HU was used as the cutoff for differentiating between benign and malignant masses. The kappa coefficient is 0.851 (95% CI: 0.78–0.922).

Alt text: Table 2 showing concordance between two observers using 10 HU as the cutoff for benign vs malignant adrenal masses, with high agreement.

### Validity of judgments

Malignant tumors, such as adrenocortical carcinomas and malignant lymphomas, as well as PCCs, were all included in the ≥10 HU group. The judgments of the two observers were discordant in 16 cases, but all involved benign pathology, supporting the notion that a CT value <10 HU reliably excludes the possibility of malignancy.

### Distribution of CT attenuation values across adrenal tumor types

**Figure 3:**
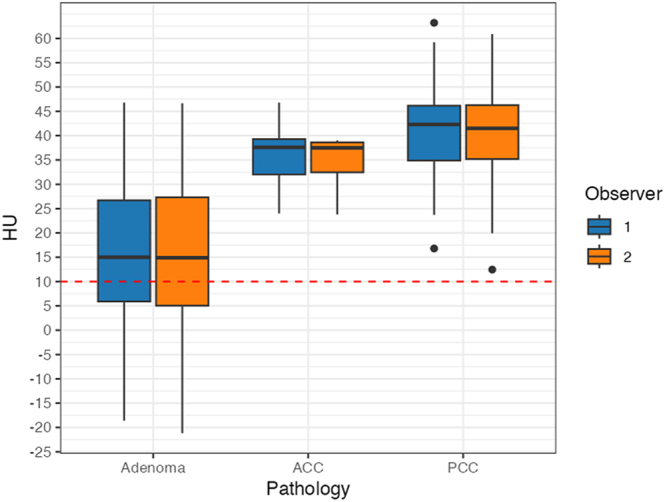
Distribution of CT attenuation values for adenomas, adrenocortical carcinomas (ACCs), and pheochromocytomas (PCCs). The boxes represent the interquartile range (IQR; 25th–75th percentile), the horizontal lines indicate medians, the whiskers extend to the most extreme values within 1.5 × IQR, and the values beyond this range are plotted as outliers. The median CT attenuation values (IQR) for adenoma, ACC, and PCC were 15.0 HU (4.6–25.4), 37.6 HU (34.0–41.2), and 42.3 HU (36.6–47.9) in observer 1 and 14.9 HU (3.8–26.1), 37.5 HU (34.4–40.6), and 41.5 HU (35.9–47.1) in observer 2, respectively. ACCs and PCCs all exhibit attenuation values >10 HU, but the distribution of values for adenomas is wide and distributed across 10 HU. Alt text: box-and-whisker plot comparing CT attenuation values (HU) for adenomas, adrenocortical carcinomas (ACCs), and pheochromocytomas (PCCs) as measured by two observers. ACCs and PCCs consistently show values above the 10 HU cutoff, whereas adenomas display a wide distribution that spans across the 10 HU threshold.

Figure 3 shows the distribution of CT attenuation values for adenomas, ACCs, and PCCs. This figure also showed that adenomas have a wider dispersion of CT attenuation values than ACCs and PCCs.

### Differences in CT attenuation values between APAs and CPAs

**Figure 4:**
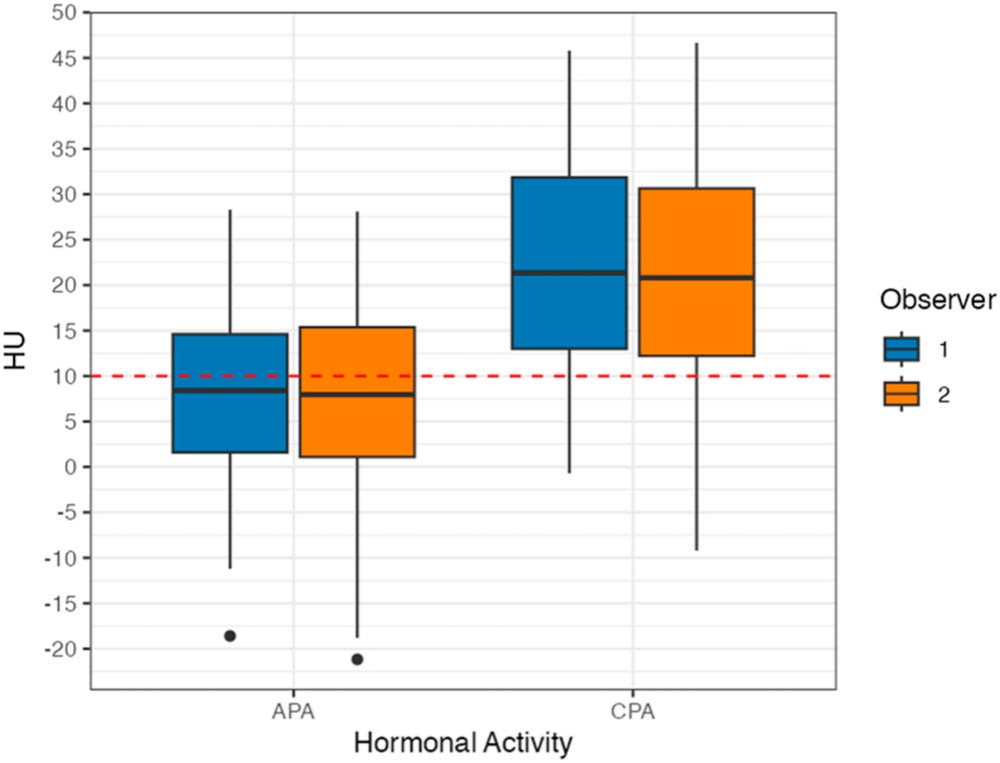
Comparison of CT attenuation values for aldosterone-producing adenomas (APAs) and cortisol-producing adenomas (CPAs). The boxes represent the interquartile range (IQR; 25th–75th percentile), the horizontal lines indicate medians, the whiskers extend to the most extreme values within 1.5 × IQR, and the values beyond this range are plotted as outliers. The median CT attenuation values (IQR) for APA and CPA were 8.40 HU (1.9–14.9) and 21.4 HU (12.0–30.9) in observer 1 and 7.95 HU (0.8–15.1) and 20.8 HU (11.6–30.0) in observer 2, respectively. In the multivariable linear regression analysis, CPAs showed significantly higher CT attenuation than APAs (observer 1: *β* = 16.3, *P* < 0.001; observer 2: *β* = 15.7, *P* < 0.001), while mass size was not significantly linked to CT attenuation (observer 1: *β* = −0.19, *P* = 0.20; observer 2: *β* = −0.18, *P* = 0.25). Alt text: box-and-whisker plot comparing CT attenuation values (HU) in aldosterone-producing adenomas (APA) and cortisol-producing adenomas (CPA). It shows that APAs are lower than CPAs, independent of mass size.

Figure 4 shows the distribution of CT attenuation values for APAs and CPAs. The CT attenuation values of APAs were lower than those of CPAs for both observers. This apparent difference remained significant even after adjustment for mass size. In the multivariable linear regression analysis, CT attenuation values measured by both observers were significantly associated with hormonal activity (overall model *P* < 0.001). CPAs demonstrated significantly higher CT attenuation than APAs (observer 1: *β* = 16.3, *P* < 0.001; observer 2: *β* = 15.7, *P* < 0.001). In contrast, mass size did not show a significant association with CT attenuation (observer 1: *β* = −0.19, *P* = 0.20; observer 2: *β* = −0.18, *P* = 0.25).

## Discussion

Our study analyzed the reliability between a general surgeon and an endocrine surgeon, obtaining an ICC of 0.938 (95% CI: 0.923–0.949), which is comparable to previous reports (13, 14). We thus showed that even surgeons can make reliable measurements by following a simple rule of placing the largest possible circular ROI without including cystic components or calcifications.

The measurement of CT attenuation values is a very simple procedure and is considered feasible even for non-radiologists. In Japan, relatively few radiologists are available for a large number of CT scanners. The number of CT scanners installed in Japan is 115.7 units per 1,000,000 people, the highest among Organisation for Economic Co-operation and Development countries (Organisation for Economic Co-operation and Development. CT scanners. Accessed December 24, 2024. https://www.oecd.org/en/data/indicators/computed-tomography-ct-scanners.html). On the other hand, the number of radiologists in Japan is small compared to other countries, and the workload is thus heavy (21). If non-radiologists could measure CT attenuation values, the burden on radiologists would be reduced. Even in countries where there are sufficient radiologists, the widespread use of measurements from non-radiologists would allow for quicker diagnosis and treatment. However, most previous studies have been performed by radiologists. Our study indicated that CT attenuation measurements remain reliable and valid even when conducted by surgeons.

Nevertheless, we identified six cases in which CT attenuation values differed substantially between the two observers, comprising CS in three cases, SCS in two cases, and APA in one case. Reexamination of these CT findings revealed the following reasons for the discrepancies: three cases had cystic components that would make it difficult to determine whether or not to avoid placing a ROI, one case had an internally heterogeneous mass that could have differed significantly depending on which slice was used, and one case had a mass that was too small in diameter to be captured as a mass between observers. In the other case, the cause was not clear. In addition, the reliability of CT attenuation values decreased when the mass diameter was ≤15 mm or >40 mm. This was presumed to be due to discrepancies among operators in identifying the mass region for small masses and discrepancies in recognizing the presence of cystic components or calcifications that should be avoided when setting the ROI for large masses. To the best of our knowledge, no reports exist regarding the relationship between adrenal mass size and the accuracy of CT attenuation value measurement. Manual measurements may have limitations in lesions with complex internal architecture and can show reduced reliability depending on lesion size.

A major strength of this study was the confirmation of both pathological diagnosis and hormonal activity in all cases. The gold standard for the diagnosis of adrenal masses is histopathology and endocrine function evaluation. However, in previous studies, these tests were not always performed in all eligible cases (3). In this study, the diagnosis of all subjects was confirmed endocrinologically and pathologically. Since all cases of adrenal malignancy and PCC diagnosed by such procedures were measured by both observers as ≥ 10 HU, with no false negatives, high validity was seen in excluding these diseases. The study is also unique in identifying differences in CT attenuation values among benign masses, particularly between APAs and CPAs. We identified a clear difference in CT attenuation value between APAs and CPAs. Although both APAs and CPAs are commonly managed surgically, the ability to estimate hormonal activity based on CT morphology may carry several clinical implications. For instance, if CT attenuation value allows us to roughly differentiate between APAs and CPAs, this may help establish a prioritization of subsequent functional tests and make the diagnostic workflow more efficient. Moreover, having some expectation based on imaging findings may facilitate smoother communication and shared decision-making with patients when explaining the necessity of biochemical evaluations. Furthermore, in primary aldosteronism, adrenal venous sampling (AVS) may be omitted when biochemical confirmation has been established and a unilateral adrenal mass is clearly identified on CT (16). In such cases, CT attenuation values consistent with the typical characteristics of APAs could provide supportive information when considering AVS omission. On the other hand, because this study was retrospective, immunohistochemical staining for CYP11B1 and CYP11B2 – techniques that were not routinely emphasized in clinical practice at the time – was not performed. In addition, in current real-world clinical practice in Japan, the differentiation of functioning adrenocortical tumors is primarily based on biochemical assessments rather than immunohistochemistry, which is not routinely used. For these reasons, we classified hormonal activity in this study according to biochemical evaluations rather than CYP11B1/2 immunostaining. Future studies investigating the relationship between hormonal activity and CT attenuation may benefit from incorporating such pathological assessments as well.

Several limitations to this study need to be kept in mind when interpreting the results. First, in real-world clinical practice, approximately half of AIs are nonfunctioning adenomas; however, only four such cases (1.3%) were included in the present study (2). Moreover, these lesions had been selected for surgery and therefore tended to represent exceptional cases with a larger mass size or higher CT attenuation value. As a result, our cohort does not adequately represent the general population of nonfunctioning adrenal adenomas. Consequently, this study might not be able to characterize typical imaging findings of nonfunctioning adenomas or to compare them meaningfully with functioning adrenal masses. Second, the two observers in this study conducted their evaluations independently under circumstances that did not allow them to refer to each other’s measurements or to the radiologist’s reading reports. In this respect, the internal validity was considered high. On the other hand, the two observers were colleagues who routinely worked at the same clinical site, and the possibility cannot be denied that procedural tendencies and criteria in measuring CT attenuation values may have been similar from the beginning. Such prior similarity may have contributed to the increased reliability between the measurements. Third, the two observers involved in this study had prior experience measuring CT attenuation values of adrenal masses in clinical practice. Therefore, the high level of interobserver reliability demonstrated in this study may not be generalizable to individuals without such experience, even if similar measurement protocols are employed. Fourth, in patients with extra-adrenal malignancy, approximately 7% of adrenal lesions with HU ≤ 10 have been reported to be malignant (3). However, adrenal metastases from extra-adrenal malignancies are often not considered surgical candidates, and such cases were therefore unlikely to be included in our study. Therefore, our findings cannot be generalized to patients with extra-adrenal malignancies. Fifth, interobserver reproducibility between non-radiologists and radiologists was not evaluated in this study; therefore, the possibility of a systematic measurement bias specific to non-radiologists cannot be completely excluded. However, because the validity of the <10 HU cutoff was assessed using pathological and endocrinological reference standards – with complete exclusion of malignant tumors and pheochromocytomas – any potential systematic bias is unlikely to be clinically meaningful in the intended diagnostic context.

In conclusion, this study demonstrated, using pathologically and endocrinologically validated cases, that CT attenuation values are a reliable and practical tool for excluding malignancy and PCCs. Importantly, this reliability was established through measurements performed by non-radiologists, suggesting that the approach could be beneficial in regions with a shortage of radiologists and could also enhance immediate clinical decision-making by surgeons and internists without the need to wait for radiologist interpretation.

## Declaration of interest

The authors declare that there is no conflict of interest that could be perceived as prejudicing the impartiality of the work reported.

## Funding

This research did not receive any specific grant from funding agencies in the public, commercial, or not-for-profit sectors.

## Data availability

The datasets generated and/or analyzed during the current study are not publicly available due to privacy restrictions but are available from the corresponding author on reasonable request.

## Ethics statement

This retrospective study was conducted in accordance with the Declaration of Helsinki and was approved by the Institutional Review Board of Tokyo Women’s Medical University (approval number 2022-0066). Owing to the retrospective design, the requirement for written informed consent was waived by the Board. Instead, information about the study was disclosed on the institutional website to provide patients with the opportunity to opt out.
